# Inflammatory Markers and Hepcidin are Elevated but Serum Iron is Lower in Obese Women of Reproductive Age

**DOI:** 10.3390/nu13010217

**Published:** 2021-01-14

**Authors:** Sixtus Aguree, Manju B. Reddy

**Affiliations:** Department of Food Science and Human Nutrition, Iowa State University, Ames, IA 50011, USA; saguree@iastate.edu

**Keywords:** serum iron, hepcidin, ferritin, BMI, inflammation

## Abstract

Limited evidence suggests that serum iron and hepcidin concentrations are dysregulated in obesity and inflammation. The objective of the present study was to compare C-reactive protein, interleukin-6, circulating levels of hepcidin, serum lipids, and iron status in obese vs. normal-weight women of childbearing age. Healthy women aged 18–30 years were recruited for the study (*n* = 47: 25 obese and 22 normal weight). Fasting blood samples were obtained to measure serum lipids (total cholesterol, HDL, LDL cholesterol, triglycerides, non-HDL cholesterol), complete blood count, serum iron, total iron-binding capacity, transferrin saturation, serum ferritin, hepcidin, C-reactive protein, and interleukin-6. Obese women had significantly higher mean serum C-reactive protein (*p* < 0.001), interleukin-6 (*p* < 0.001), hepcidin (*p* = 0.024), triglycerides (*p* < 0.001) and total cholesterol/HDL ratio (*p* < 0.001) but lower HDL (*p* = 0.001) and serum iron/hepcidin ratio (*p* = 0.011) compared with normal-weight women. BMI correlated positively with inflammatory markers, triglycerides, LDL and total cholesterol/HDL ratio, and negatively with HDL and serum iron/hepcidin ratio. Serum iron correlated negatively with ferritin in the obese group (*p* = 0.030) but positively in normal weight women (*p* = 0.002). BMI and ferritin were the only predictors of serum iron/hepcidin ratio accounting for 23% of the variation among subjects. Studies are needed to examine anti-inflammatory dietary approaches that can improve iron biomarkers in obese women.

## 1. Introduction

Obesity and iron deficiency anemia are the two leading public health problems globally. More than one in three adults worldwide are obese [1,2], and anemia affects 1.95 billion people [3], with approximately 50% of the cases attributed to iron deficiency (ID) [4,5]. Multiple iron indices are recommended when assessing iron status, and is particularly complicated under inflammatory conditions such as obesity. The World Health Organization (WHO) and Centers for Disease Control and Prevention (CDC) recommends measuring serum ferritin and soluble transferrin receptor as the best approach for assessing iron status of a population [6]. However, serum ferritin concentration is elevated in the presence of inflammation, which can lead to underestimation of iron deficiency in a population [7,8]. Many approaches have been proposed for adjusting for the effect of inflammation on ferritin and iron markers [9,10] but these often lead to different estimates of population iron status [11,12]. This makes iron status assessment difficult, particularly in areas with endemic infection (e.g., malaria) and inflammation [13,14].

Recently, there has been a considerable research interest on hepcidin as an indicator of iron status and to identify iron deficiency in a population [15,16]. Hepcidin binds to ferroportin—a transmembrane protein that is involved in cellular efflux of iron, at the basolateral membrane of the enterocyte, macrophage, and hepatocyte—followed by its internalization and degradation [17]. Consequently, increased hepcidin concentration is linked to reduced intestinal iron absorption and decreased iron release from body stores, resulting in low serum iron [18,19,20]. In healthy persons with iron deficiency and iron deficiency anemia (IDA), serum hepcidin, ferritin, and transferrin saturation are significantly low [21,22]. However, just like ferritin, hepcidin concentration is elevated in inflammatory conditions such as obesity.

Thus, the rising level of obesity has significant public health implications on iron status because of increased adiposity, which induces inflammation [23,24] and raises levels of pro-inflammatory cytokines in systemic circulation [25,26]. Elevated cytokine concentration stimulates the hepcidin gene expression and increases hepcidin concentration [27,28]. Thus, obesity-associated low-grade inflammation may induce low circulating serum iron and increases the risk of iron deficiency anemia through hepcidin-mediated reduced iron absorption and release from storage [29]. Irondeficiency disproportionally affects preschool-age children and women of reproductive age [5,30], but few studies have simultaneously examined inflammation and iron status of young obese women [31]. Evaluation of several iron status indices and inflammation markers in healthy obese compared with normal-weight women of similar age would provide insight in understanding iron status in the non-pregnant state. Previously, Stoffel et al. [31] evaluated different iron and inflammatory biomarkers in normal-weight vs. obese women. They found higher androidal fat mass (central adiposity) correlated with elevated C-reactive protein (CRP), α-1 glycoprotein (AGP), serum hepcidin, and total iron-binding capacity (TIBC), and lower serum iron/hepcidin ratio (SFe/Hep) and transferrin saturation (TSAT) [31]. The present study examined the influence of obesity on inflammatory markers, iron status indicators, and serum lipids, in healthy women of childbearing age—obese vs. normal-weight. Our hypothesis was that serum iron concentration will be lower in obese women because of elevated hepcidin concentrations.

## 2. Materials and Methods

### 2.1. Study Design

This cross-sectional study evaluated inflammatory markers, iron status indicators, and serum lipids among women of childbearing age (obese women, BMI: ≥30 kg/m^2^; normal weight, BMI: 18.5 to 24.9 kg/m^2^). Participants were recruited from Iowa State University by mass email and flyers posted on campus from November 2019 to March 2020. Inclusion criteria were: female, 18–44 years of age, generally good health (does not have a known, ongoing health condition/medical issue that requires regular monitoring by a physician or regular visits to the hospital), nonsmoking, and non-pregnant. Participants were excluded if they had any of the following conditions: blood pressure (systolic blood pressure (SBP) < 90 or ≥130 mmHg and/or DBP < 60 or ≥80 mmHg), chronic hypertension, or previous hypertensive disorder in pregnancy (gestational hypertension or preeclampsia), gastrointestinal problem or diabetic. Interested persons completed an online screening questionnaire via Qualtrics ^TM^ (Qualtrics, Provo, UT, USA). Eligible subjects made two study visits; in the first visit, weight (to the nearest 0.1 kg) and height (to the nearest 0.1 cm) were measured at the Nutrition and Wellness Research Center at Iowa State University to confirm eligibility. A trained study staff measured standing height (Model S100; Ayrton Corp., Prior Lake, MN, USA), weight (Abco Health-o-meter; Bridgeview, IL, USA) to calculate BMI. In the second visit, blood samples were collected from the eligible participants (for those with BMI confirmed) after overnight (10 h) fasting, which occurred between 7 and 9 a.m. for biochemical measurements described below. Blood samples were centrifuged at 1300× *g* for 15 min (4 °C) to collect serum and frozen at −80 °C until the end of the study. The study protocol was approved by the Institutional Review Board, Office of Research Ethics at Iowa State University, and participating women signed an informed consent form online.

### 2.2. Biochemical Measurements

Blood samples were analyzed by a certified clinical laboratory (Quest Diagnostics, Chicago, IL, USA) for blood chemistry analysis, including serum iron and other iron status markers and lipid panel (total cholesterol (TC), high-density lipoprotein (HDL), low-density lipoprotein (LDL), triglycerides, non-HDL cholesterols). Serum was used to measure ferritin (Ramco. Laboratories Inc., Stafford, TX, USA), hepcidin [Hepcidin 25 (Bioactive); DRG International, Inc., Springfield, NJ, USA], serum CRP (high-sensitivity kit, ALPCO Diagnostics, Salem, NH, USA), and interleukin-6 (IL-6) (High Sensitive Kit, Cambridge, MA, USA) using a microplate reader (Bio-Tek Instruments Inc., Winooski, VT, USA). All measurements were performed in duplicates. The intra-assay CVs for ferritin, Hepcidin, IL-6, and CRP, were 6.6%, 8.1%, 9.9%, and 4.3%, respectively.

### 2.3. Statistical Analysis

Categorical descriptive variables were presented as frequencies (%). Continuous variables with normal distribution were presented as mean ± SD unless otherwise stated. Normality was assessed via the Shapiro–Wilk test and by visual inspection of kernel density plots. The data for ferritin, hepcidin, CRP, and IL-6 were skewed and therefore were log-transformed before statistical analysis and presented as geometric means. We used Fisher’s exact test for categorical variables and independent one-sided *t*-tests to compare the differences in normally distributed variables. A Spearman correlation coefficient was computed to assess the relationship between BMI and each biomarker. Our primary outcome was the difference in serum iron concentration between obese and normal-weight women. We hypothesized that obese women would have lower serum iron concentration than normal-weight women. With a mean difference of 19.6 µg/dL and a pooled standard deviation of 38.5 µg/dL, our data have a statistical power of 77% to detect differences in serum iron concentration between the two groups.

We used stepwise multiple regression models to examine variables that predict serum SFe/Hep. We ran two models, first with all subjects included, and second with obese women only. The first model included age, BMI, HDL, triglycerides, and ferritin as independent variables and SFe/Hep as the dependent variable. The second model included the same variable except that CRP was used in place of BMI. Whether BMI was used as a categorical (obese vs. normal) or as a continuous variable did not change the interpretation of results. All statistical analyses were conducted using Stata Version 14 software (Stata Corp, College Station, TX, USA).

## 3. Results

### 3.1. Characteristics of Study Participants

**Figure 1:**
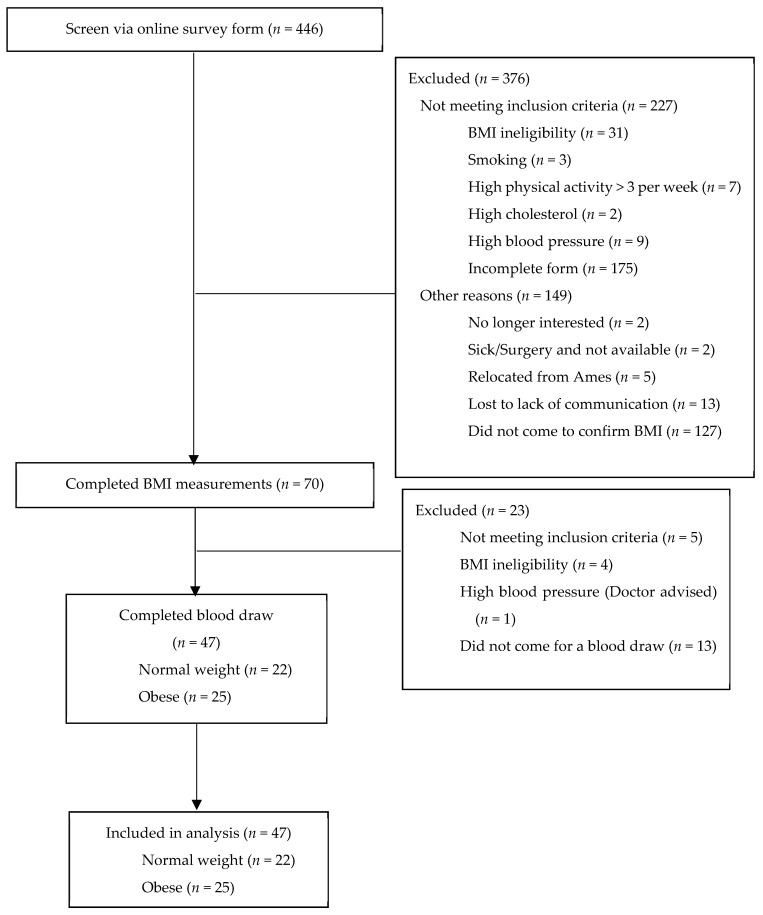
Study design and eligibility of participants.

We screened 446 women via an online screening questionnaire, of which 70 were eligible based on their weight and height measured to confirm their BMI. Eventually, 47 women (25 obese and 22 normal weight) completed the blood draw and were included in this analysis (Figure 1).

The mean age of the subjects was 21.0 ± 2.9 years (range; 18–30). The majority (61.7%) of the women reported that their last three menstrual cycles were regular (26–35 days)—obese women 36.0% (irregular cycle) vs. 64% (regular cycle) and for normal-weight women 40.9% (irregular cycle) vs. 59.1% (regular cycle). Similar numbers of participants (63.8%) reported currently using a hormonal birth control (BC) method (e.g., Mirena, intrauterine devices, Depo-Provera—“the shot”, “the pill”)—obese women 48.0% (non usersBC) vs. 52.0% (use BC) and for normal-weight women 22.7% (non users-BC) vs. 77.3% (use BC). There were no differences in iron biomarkers between those who use BC and those not using any BC method. Participants were young, predominantly nulliparous (97.9%), and mostly white non-Hispanic undergraduate students (Table 1). A little over a third (34.0%) (36.0% obese vs. 31.8% normal-weight) of the women reported taking micronutrient supplements during the survey. There were no significant differences in iron markers between women who reported taking the supplements or not. Four were anemic according to the WHO criteria (hemoglobin < 12 g/dL) [32] (three in obese and one in the normal-weight group). Obese women had lower mean corpuscular volume (MCV) (obese (*n* = 5, 20.0%) vs. normal-weight (*n* = 2, 9.0%)) and mean corpuscular hemoglobin concentration (MCHC) (obese (*n* = 3, 12%) vs. normal-weight (*n* = 0, 0.0%)), less than 80 fL and 32 g/dL, respectively. Women underreported their weight: normal-weight women by a mean difference of 3.1 ± 12.4 kg (56.9 ± 14.1 kg self-reported vs. 60.0 ± 7.3 kg measured), and obese women by a mean difference of 1.3 ± 3.7 kg (100.2 ± 17.6 kg self-reported vs. 101.4 ± 17.6 kg measured).

### 3.2. Biochemical Indices

Our study population was healthy women, generally non-anemic, with normal ferritin concentration (values averaging 34–38 ng/mL). All markers of inflammation, IL-6, and CRP, including white blood cells count, were significantly higher in obese women (*p* < 0.001) when compared to normal-weight women. Red blood cell indices, including hemoglobin, hematocrit, and mean corpuscular hemoglobin (MCH), were similar between groups, while MCV appears to trend towards lower in obese women. Serum iron concentration was lower in obese women (*p* = 0.044), but TIBC, TSAT, and ferritin were similar between the two groups (*p* > 0.050). As expected, obese women had significantly higher serum hepcidin concentration (*p* = 0.024) but lower SFe/Hep (*p* = 0.011) compared to normal-weight women. Mean TC concentration was comparable between normal-weight and obese women (*p* > 0.050) (Table 2). Compared to normal-weight, HDL concentration was 20% lower, while triglycerides and TC/HDL ratios were 38% and 32% higher in the obese group, respectively (all *p* < 0.05).

As expected, we found a strong positive correlation between BMI and CRP (*n* = 39, r = 0.57, *p* < 0.001), IL-6 (*n* = 40, r = 0.40, *p* = 0.010), triglycerides (*n* = 47, r = 0.44, *p* = 0.002), and LDL (*n* = 47, r = 0.33, *p* = 0.025), but negative correlation with HDL (*n* = 47, r = −0.53, *p* < 0.001) and SFe/Hep (*n* = 40, r = −0.39, *p* = 0.012) (Table 3). However, BMI was moderately correlated with hepcidin (*n* = 40, r = 0.30, *p* = 0.064) and serum iron concentration (*n* = 45, r = −0.28, *p* = 0.063) individually. As predicted, serum iron correlated negatively with ferritin in the obese group (*n* = 22, r = −0.46, *p* < 0.030) but positively in normal weight women (*n* = 20, r = 0.65, *p* = 0.002) (data not shown).

Based on the regression analysis including all subjects, BMI and ferritin were the only predictors of SFe/Hep—here data were combined for both obese and normal-weight subjects to determine the relationship between predictor variables and the outcome of interest. A unit increase in ferritin was associated (borderline significant) with 7.04 units decline in SF/Hep, when controlled for BMI. Age, HDL, and triglycerides were included in the models but were not significant. Together BMI and ferritin accounted for 23% of SFe/Hep variations among participants (adjusted R^2^ = 19%). Among obese women, CRP and ferritin were the only predictors of SFe/Hep, and accounted for 33% of the variation in SFe/Hep (adjusted R^2^ = 26%) (Table 4).

## 4. Discussion

Previous studies in premenopausal obese women have reported a strong link between increased adiposity and inflammation (CRP, IL-6, AGP) [31,33] and serum hepcidin concentration [20,34]. In the present study, we found that BMI was positively associated with serum CRP, IL-6, and hepcidin concentrations—with concentrations two to eight times higher in obese subjects. These findings are expected since increase adiposity is directly linked to inflammation [23], and obesity and inflammation are independently associated with higher hepcidin concentrations [35,36,37]. Obesity induces pro-inflammatory cytokines, which in turn causes increase hepcidin concentration. This occurs because increased adiposity promotes the proliferation of macrophages and low-grade inflammation [24,26,38]. The accumulation and activation of macrophages release cytokines into the systemic circulation [24,25], particularly IL-6 (but also IL-1α and IL-1β), which stimulates hepatic hepcidin expression [39,40] and increased serum hepcidin concentration [27]. Excess hepcidin increases endocytosis and proteolysis of ferroportin, keeping iron trapped within cells. This reduces iron efflux from hepatocyte, duodenal enterocytes, macrophages resulting in reduce plasma iron concentration [17].

Though serum ferritin concentration was similar in the two groups, the relationship between serum iron and ferritin was different in obese and normal-weight women. Higher serum iron correlated positively with higher ferritin in normal-weight subjects whereas in obese women, a negative correlation was observed. We suspect that the elevated inflammatory markers and hepcidin observed in the obese group downregulated the serum iron concentration, which was not observed in the normal-weight subjects. The increase in hepcidin concentration in the obese group may be in response to elevated IL-6 concentration, as reported in previous studies [39]. Since higher hepcidin is related to low serum iron concentration, we combined the two markers into a single index, SFe/Hep, to provide a better understanding about iron status. Having higher hepcidin and lower serum iron concentrations in the high BMI group supported our hypothesis. Our findings of lower circulating serum iron in obese women is in agreement with previous well-controlled studies [41], thus providing evidence of decreased serum iron concentration in obesity.

A recent study among women of comparable age to the present study reported higher hepcidin, CRP, IL-6, but lower SFe/Hep in obese women compared with normal weight women [31]. These findings are consistent with our results. We found a small but significantly lower serum iron and SFe/Hep in obese women suggesting altered iron distribution, which can be attributed to serum hepcidin-induced sequestration of iron in obesity, causing reduced iron export from macrophages, enterocytes, and hepatocytes [42,43]. This is supported by the negative correlation between serum iron and ferritin in obese women, indicating that though they had a normal ferritin level, the iron released from hepatocytes was limited compared to normal weight women. Overall, BMI and ferritin explained 23% of the SFe/Hep variations in the study population. Among obese women, inflammation (indicated by CRP) and ferritin explained a third of the SFe/Hep variation. The negative association between CRP and SFe/Hep provides further support for the significant influence of inflammation in suppressing serum iron concentration in apparently healthy obese individuals. Even when dietary iron absorption was similar in obese and normal-weight women, the SFe/Hep was significantly higher in the former [31]. The implications of our findings in terms of an intervention addressing iron deficiency in obesity is that researchers should pay attention to reducing inflammation—a common underlying issue in obesity.

Studies have demonstrated a consistent and robust link between increased adiposity and dyslipidemia. For instance, we have previously reported that centrally located body fat predicted cardiometabolic risk, including lower HDL, elevated LDL and triglycerides, and CRP concentrations in healthy postmenopausal women [44,45] and was corroborated by other researchers [46,47]. The similarities in findings provide further evidence that increased adiposity is a significant predictor of dyslipidemia and inflammation, which are risk factors for cardiometabolic problems even in healthy premenopausal women. Our results suggest that increased inflammation mediates the link between adiposity and dyslipidemia. Reducing adiposity can improve cardiometabolic health because of the associated changes in serum lipids and inflammatory markers. For instance, six months after bariatric surgery in young and middle-aged women, authors reported a significant reduction in triglycerides, inflammatory markers, and TC but an increase in HDL [18,48,49].

An increase in BMI has been associated with higher blood volume. It is speculated that the alteration in iron biomarkers is in response to a demand to meet the need for increase erythropoiesis [41]. However, more empirical work is needed to clarify this relationship. Our findings support the growing literature showing that obesity-associated inflammation induces dysregulation in iron biomarkers via elevated hepcidin concentration. Future studies will be needed to clarify the effect of reducing inflammation with or without a reduction in adiposity on serum hepcidin concentrations and iron status among obese individuals. The differences in IL-6, hepcidin and serum iron between obese and normal-weight were marginally small but statistically significant, and the mean values for both groups are within normal range for all three biomarkers [7,50]. The CRP values in the obese group were 8 times higher than normal-weight women—most of the normal weight subjects were within normal range, those of the obese group were elevated. It is possible that some of the participants had other pathologies related to chronic disease of inflammation that were unknown [51]. We suspect that these differences in IL-6 observed in the present were related to obesity since IL6 values are about 3 times higher as reported in a previous study with rheumatoid patients [52]. Though differences in these biomarkers between the two groups were small, we believe that these are biologically relevant and represent an early biomarker of larger changes in the future given that the mean BMI among obese women was 36.7 ± 6.0 kg/m^2^ (more than half with BMI < 35), as CRP, and IL-6 both increased with increased adiposity, as has been previously reported [18].

Other areas that should be explored in the future include quantifying the influence of the menstrual cycle and the use of hormonal birth control on iron biomarkers within the context of obesity. For instance, the menstrual cycle has been reported to impact systemic iron homeostasis including hepcidin and serum iron [53] but blood collection for this study was not timed to the cycle phase. It is unknown how much of the differences or lack thereof in iron markers in the present study could be attributed to the time within the menstrual cycle blood samples were collected. Furthermore, more than half of the participants reported using hormonal contraceptives, which may be a potential confounder affecting iron biomarkers observed in the current study. In two large studies from Tanzania and the US, both menstrual cycle and the use of hormonal contraceptives were associated with iron biomarkers. In this large cross-sectional study in Tanzania (*n* = 4186 women), the prevalence of ID was high (30%) but only one in five (19%) women reported history of hormonal contraceptive use, and history of hormonal contraceptive use was negatively associated with ID, IDA, and anemia, after controlling for potential confounders [54]. Similar findings have been reported in the US based on the analysis of the 1999–2006 National Health and Nutrition Examination Survey. In this study, the ID prevalence was 9.8% and 77.5% of women reported ever using hormonal contraceptive. Furthermore, ferritin and percent transferrin saturation were negatively associated with having regular menstrual periods while transferrin receptor levels were positively associated with having regular menstrual periods. The use of hormonal contraceptive was also positively associated with ferritin but negatively with the transferrin receptor [55]. In the present study, we did not find any statistically significant difference in iron biomarkers between women who reported using or not using hormonal birth control methods.

There are a number of strengths and limitations to this research. Measurement of iron absorption and ferroportin concentration could provide important information as to whether they were associated with higher and lower serum iron concentration in this population. Despite this limitation, the findings of this study are important because we measured a range of physiological factors that can affect iron status and cardiovascular health, thus, providing a bigger picture of metabolic dysregulation occurring in obesity.

## 5. Conclusions

The current study results showed that increased adiposity in young, healthy obese women is associated with elevated serum hepcidin, inflammation, dyslipidemia, and depressed serum iron concentrations. However, more extensive studies are needed to explore the consequences associated with this relationship. Future studies should examine if dietary approaches to reduce inflammation improve cardiovascular indices and iron biomarkers, including ferritin, hepcidin, and serum iron concentrations in obese women.

## Figures and Tables

**Table 1:** Characteristics of study participants (normal weight and obese women) (*n* = 47).

Variable	Normal Weight (BMI: 18.5–24.9 kg/m^2^)	Obese (BMI: >29.9 kg/m^2^)	*p* Value
	Mean ± SD	Mean ± SD	
N	22	25	
Age (years)	21.5 ± 3.4	20.6 ± 2.3	0.307
Height (cm)	166.4 ± 6.8	166.1 ± 5.6	0.862
Weight (kg)	60.0 ± 7.3	101.4 ± 17.6	<0.001
BMI (kg/m^2^)	21.6 ± 1.6	36.7 ± 6.0	<0.001
Race, *n* (%)			0.693
White non-Hispanic	16 (72.7)	17 (68.0)	
Black/African American non-Hispanic	1 (4.5)	0 (0.0)	
Hispanic	2 (9.0)	6 (24.0)	
Asian	2 (9.0)	1 (4.0)	
Multiple or others	1 (4.5)	1 (4.0)	
Parity, *n* (%)			0.468
0	21 (95.5)	25 (100.0)	
1	1 (4.5)	0 (0.0)	
Educational status, *n* (%)			0.177
At least a bachelor’s degree	5 (22.7)	4 (16.0)	
Associate degree	3 (13.6)	2 (8.0)	
Undergraduate students	14 (63.6)	19 (76.0)	

BMI, body mass index, *p*-values for age, height, and BMI were obtained by independent *t*-test (two-sided) and for race, parity, and educational status by Fisher’s exact test.

**Table 2:** Inflammatory markers, iron status, and serum lipid concentrations in obese (*n* = 25) and normal weight healthy young women (*n* = 22).

Variable	Normal Weight Women (BMI: 18.5–24.9 kg/m^2^)	Obese Women (BMI: >29.9 kg/m^2^)	*p* Value
	Mean ± SD	Mean ± SD	
Inflammatory markers			
WBC (X 10^3^/µL)	5.6 ± 1.6	7.7 ± 2.0	<0.001
IL-6 (pg/mL) ^1^*	1.46 [1.13, 1.89]	2.16 [1.86, 2.51]	0.003
CRP (mg/L) ^1^*	8.2 [3.1, 21.8]	69.9 [41.1, 118.9]	<0.001
Iron biomarkers			
Hemoglobin (g/dL)	13.6 ± 1.1	13.3 ± 1.1	0.166
Hematocrit (%)	39.4 ± 2.8	39.2 ± 2.5	0.410
MCV (fL)	86.3 ± 4.5	82.4 ± 8.9	0.030
MCH (pg)	29.8 ± 2.1	30.4 ± 12.5	0.407
MCHC (g/dL)	34.5 ± 0.9	33.9 ± 1.5	0.039
Serum iron (µg/dL) ^2^	112.0 ± 41.4	92.4 ± 33.9	0.044
TIBC (µg/dL) ^2^	394.4 ± 61.5	371.3 ± 50.4	0.086
TSAT (%) ^2^	29.7 ± 13.3	25.2 ± 9.1	0.094
Ferritin (ng/mL) ^1^*	34.0 [21.0, 55.1]	37.7 [26.9, 52.9]	0.355
Hepcidin (ng/mL) ^1^	6.21 [4.39, 8.77]	11.21 [7.04, 17.83]	0.024
Serum iron/Ferritin ^2^*	3.02 [2.17, 4.20]	2.24 [1.44, 3.47]	0.134
SFe/Hep ^2^*	15.63 [11.35, 21.51]	7.53 [4.49, 12.63]	0.011
Serum Lipids			
TC (mg/dL)	170.1 ± 29.6	176.0 ± 40.1	0.288
HDL (mg/dL)	61.6 ± 12.0	49.3 ± 14.1	0.001
Triglycerides (mg/dL)	88.0 ± 29.5	121.8 ± 32.1	<0.001
LDL (mg/dL)	90.6 ± 27.5	104.2 ± 34.1	0.072
TC/HDL Ratio	2.8 ± 0.7	3.7 ± 1.0	<0.001
Non-HDL (mg/dL)	108.5 ± 28.3	126.7 ± 37.6	0.035

WBC, White Blood Cell Count; CRP, C-reactive protein; IL-6, interleukin-6, MCV, mean corpuscular volume (mean cell volume); MCH, mean corpuscular hemoglobin; MCHC, mean corpuscular hemoglobin concentration; TIBC, total iron-binding capacity; TSAT, transferrin saturation; SFe/Hep, serum iron/hepcidin ratio; TC, total cholesterol; HDL, high-density lipoproteins, and low-density lipoproteins. ^1^ Data available for 40 women (18 normal weight vs. 22 obese); ^2^ Data available for 42 women (20 normal weight vs. 22 obese); * Geometric mean (95% CI).

**Table 3:** Correlation of BMI with inflammatory markers, iron status, and serum lipid concentrations in healthy young women (*n* = 47) ^1^.

	R	*p* Value
Inflammatory markers		
CRP (mg/L) ^2^	0.57	<0.001
IL-6 (pg/mL) ^2^	0.40	0.010
Iron biomarkers		
Hemoglobin (g/dL)	−0.14	0.353
Hematocrit (%)	−0.04	0.780
MCV (fL)	−0.29	0.049
MCH (pg)	−0.05	0.726
MCHC (g/dL)	−0.22	0.138
Serum iron (µg/dL)	−0.28	0.063
TIBC (µg/dL)	−0.20	0.186
TSAT (%)	−0.22	0.139
Ferritin (ng/mL) ^2^	0.09	0.580
Hepcidin (ng/mL) ^2^	0.30	0.064
Serum iron/SHep	−0.39	0.012
Serum Lipids		
TC (mg/dL)	0.14	0.332
HDL (mg/dL)	−0.53	<0.001
Triglycerides (mg/dL)	0.44	0.002
LDL (mg/dL)	0.33	0.025
TC/HDL Ratio	0.63	<0.001
Non-HDL (mg/dL)	0.37	0.011

WBC, White Blood Cell Count; CRP, C-reactive protein; IL-6, interleukin-6, MCV, mean corpuscular volume (mean cell volume); MCH, mean corpuscular hemoglobin; MCHC, mean corpuscular hemoglobin concentration; TIBC, total iron-binding capacity; TSAT, transferrin saturation, TC, total cholesterol; HDL, high-density lipoproteins, and low-density lipoproteins. ^1^ A total for 47 women except for serum iron, TIBC, and TSAT (*n* = 45); ferritin (*n* = 42); IL-6, hepcidin, and SFe/Hep (*n* = 40); and CRP (*n* = 39). ^2^ analysis was performed on log values.

**Table 4:** Predictors of SFe/Hep in reproductive age women (Model 1, normal-weight and obese women combined; Model 2, obese women only) ^1^.

Model ^2^	Independent Variables	Likelihood Ratio ^3^	Parameter Estimate	SE	*t* Value	*p* > |t|
Model 1						
SFe/Hep		3.92 (0.048)		F (2,37) = 5.67, *p* = 0.0071, R-squared = 23%, Adj_R-squared = 19%		
	BMI (kg/m^2^)		−0.40	0.16	−2.51	0.017
	Ferritin (ng/mL) ^2^		−7.04	3.6	−1.95	0.058
	Intercept		37.78	7.12	5.30	<0.001
Model 2						
SFe/Hep		8.74 (0.013)		F (2,19) = 4.63, *p* = 0.023, R-squared = 33%, Adj_R-squared = 26%		
	CRP (mg/L)		−13.00	4.85	−2.68	0.015
	Ferritin (ng/mL)		2.67	1.34	2.00	0.060
	Intercept		20.71	8.60	2.41	0.026

BMI, body mass index; HDL, high-density lipoproteins; LDL, low-density lipoproteins; SFe/Hep, Serum iron/hepcidin ratio; CRP, C-reactive protein. ^1^ The number of observations included in the analysis varied depending on the outcome: Data available for 42 women (20 normal weight vs. 22 obese) for SFe/Hep, data available for 40 women (18 normal weight vs. 22 obese) for CRP. ^2^ Regression analysis for overall sample (Model 1): The based model included age and BMI. The full model included additional covariates: HDL, triglycerides, and log ferritin, with SFe/Hep as dependent variable. Model 2 (included only obese subjects): The based model included age and log CRP. The full model included additional covariates: HDL, triglycerides, and log ferritin, with SFe/Hep as dependent variable. ^3^ The likelihood ratio for each model represents the ratio of the full model to the based model. *p* values are in parentheses.

## Data Availability

The data presented in this study are available on request from the corresponding author. A data sharing agreement will be requested.
